# Study on initiating approach of (3-(tert-butylperoxy)propyl) trimethoxysilane on the polymerization of acrylonitrile as an initiator[Fn FN0001]

**DOI:** 10.1080/15685551.2017.1364031

**Published:** 2017-08-23

**Authors:** Yazhen Wang, Yutao Di, Shaobo Dong, Zijian He, Xueze Zhang, Jia Shi, Tianyu Lan

**Affiliations:** ^a^ Department of Materials Science and Engineering, Qiqihar University, Qiqihar, China; ^b^ Department of Chemistry and Chemical Engineering, Qiqihar University, Qiqihar, China

**Keywords:** (3-(tert-butylperoxy)propyl) trimethoxysilane, polymerization of AN, redox initiation system, modified nano-TiO_2_

## Abstract

(3-(tert-butylperoxy)propyl) trimethoxysilane (TBPT), is a tailor-made new style silane coupling agent with peroxide group, which have ability of initiating polymerization. This study used TBPT to generate free radical, and initiated the polymerization of acrylonitrile (AN), thereby forming polyacrylonitrile (PAN) in two approaches, thermal initiation system and redox initiation system. Meanwhile this study bonded TBPT onto nano-TiO_2_ to get modified nano-TiO_2_ by means of the coupling function of TBPT, and then made the peroxide group of the modified nano-TiO_2_ decompose and initiate the polymerization of AN in thermal initiation system and redox initiation system respectively. The products were investigated and analyzed by FTIR, XPS and TG. The result showed that on one hand, in the products of the thermal initiation there was PAN, which both attached and unattached to the modified nano-TiO_2_; on the other hand, in the products of the redox initiation system the PAN unattached to the modified nano-TiO_2_ was produced, while the PAN attached to the modified nano-TiO_2_ was not.

## 1. Introduction

Silane coupling agent is a category of organosilicon compounds containing different functional groups in the molecule. The silane coupling agent is able to combine with both inorganic materials and organic materials by the chemical reaction, and also it can be used for surface treatment [1–4]. Moreover, in order to obtain inorganics grafted with polymer, besides the coupling agent the initiator is also essential [5,6]. Liqun Ma et al. [7] modified nano-TiO_2_ by using vinyl (tri-tert butyl)peroxy silane as modifying agent, and made a polyacrylonitrile (PAN) layer on nano-TiO_2_ surface by using BPO as an initiator.

3-(tert-butylperoxy)propyl trimethoxysilane (TBPT) is a silane coupling agent synthesized by our laboratory [8]. It can generate free radicals by heating and initiate free-radical polymerization [9–11]. A redox initiation system can be formed when Fe^2+^ is added into TBPT, this formed redox initiation system can initiate the polymerization at a lower temperature. There are two free radicals were produced by the peroxide group of TBPT when heated. However, there is only one free radical could be produced from peroxide group in redox initiation system [12–14]. Miklos Czaun et al. [15] presented the synthesis of a novel family of surface attachable radical initiators derived from 4,4-azobis(4-cyanopentanoic acid) (ACPA). The synthetic process consists of the esterification reaction of ACPA with 10-undecen-1-ol followed by hydrosilylation of the carbon-carbon double bonds. The new initiators have been immobilized onto silica particles as initiators for the surface-initiated polymerization of both water soluble and water insoluble vinyl monomers such as N-vinyl pyrrolidone and styrene.

In this study, TBPT was used to initiate the polymerization of acrylonitrile (AN) in thermal initiation system and redox initiation system respectively. In addition, TBPT and nano-TiO_2_ were used to prepare modified nano-TiO_2_. The issues of both TBPT and modified nano-TiO_2_ initiating the polymerization of AN by thermal initiation system and redox initiation system were respectively discussed.

## 2. Experiment

### 2.1. Materials

TBPT (previously prepared), N, N-Dimethylformamide (DMF), toluene, acetone, ferrous chloride (FeCl_2_), rutile type nano-TiO_2_ VK-T80 (80 nm), AN, sodium bicarbonate (NaHCO_3_), methyl alcohol and absolute ethyl alcohol supplied by Aladdin Industrial Corporation (Shanghai, China). All of the chemicals are analytical reagent.

### 2.2. Polymerization of AN initiated by TBPT

#### 2.2.1. Polymerization of AN initiated by TBPT in thermal initiation system

**Scheme 1.**
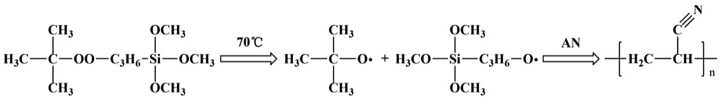
Polymerization of AN initiated by TBPT in thermal initiation system.

The deionized water and AN were added by a definite ratio into a three-neck-flask, stirred at 70 °C for 15 min under purified N_2_ atmosphere. Ethanol-TBPT solution was added into the three-neck-flask. The polymerization reaction carried out for 6 h. The crude product was refined and washed by methyl alcohol, and then dried under vacuum at 60 °C for 24 h. The experiment process was shown as Scheme 1.

#### 2.2.2. Polymerization of AN initiated by TBPT in redox initiation system

**Scheme 2.**
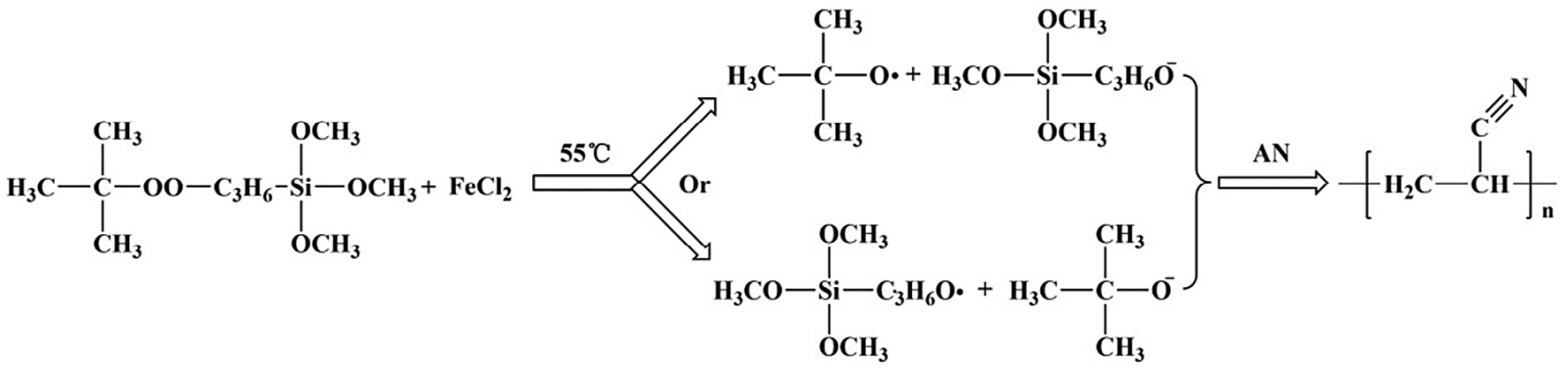
Polymerization of AN initiated by TBPT in redox initiation system.

The deionized water and AN in a definite ratio were added into a three-neck-flask, stirred at 55 °C for 15 min under purified N_2_ atmosphere. Ethanol-TBPT solution and aqueous solution of ferrous chloride were respectively added into the three-neck-flask. The polymerization reaction carried out for 6 h. The crude product was refined and washed by methyl alcohol, then dried under vacuum at 60 °C for 24 h. The experiment process was shown as Scheme 2.

### 2.3. Polymerization of AN initiated by modified nano-TiO_2_

#### 2.3.1. The preparation of modified nano-TiO_2_

**Scheme 3.**
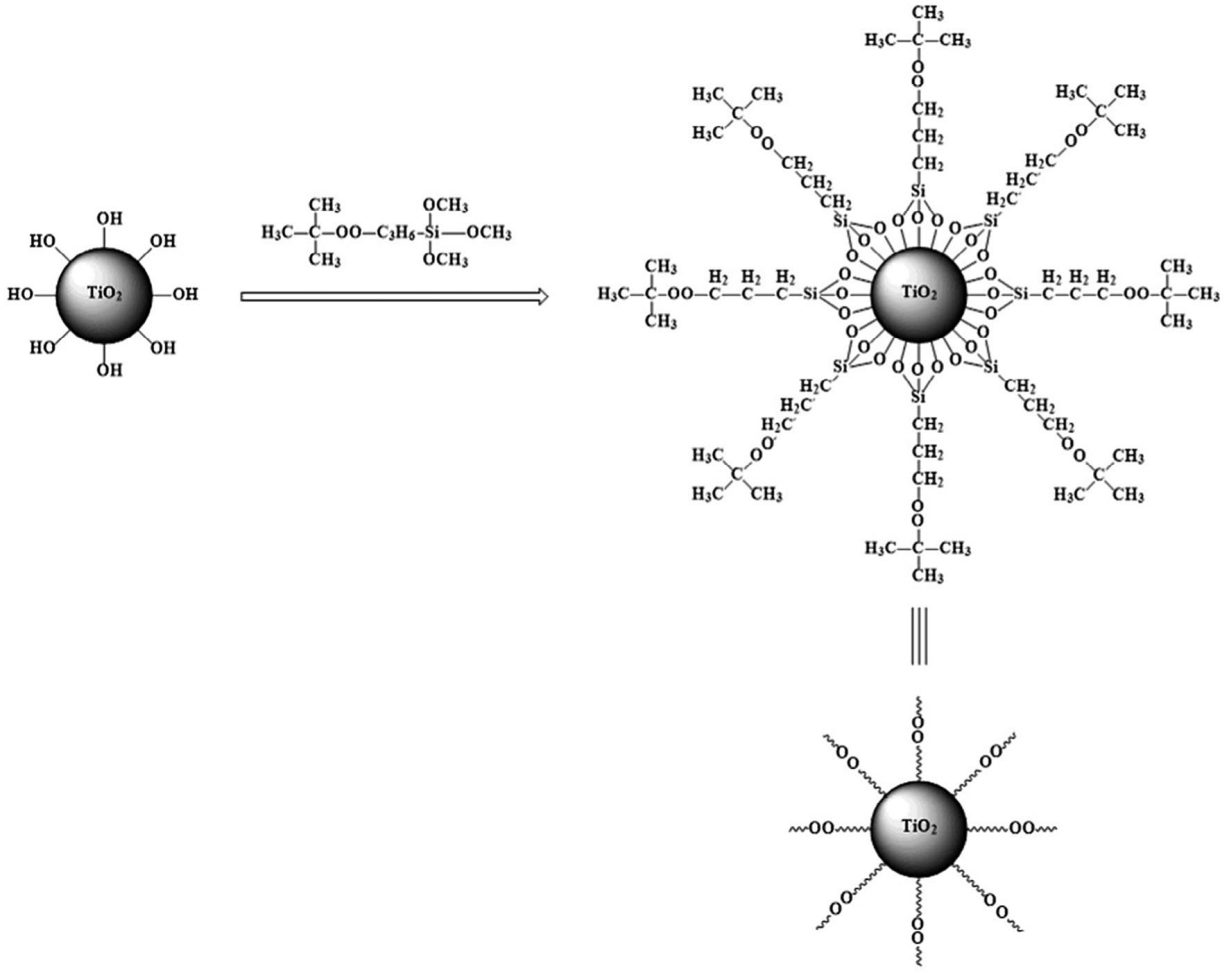
The preparation of modified nano-TiO_2_.

Nano-TiO_2_ and methyl alcohol-water solution were added into a three-neck-flask. NaHCO_3_ (10 wt%) was used to adjust the pH of the solution to 9. And then TBPT was added, stirred at 40 °C for 5 h under purified N_2_ atmosphere. Later the precipitate was washed by acetone so as to remove the unreacted TBPT. At last modified nano-TiO_2_ [7] was collected by centrifugation. The experiment process was shown as Scheme 3.

#### 2.3.2. Polymerization of AN initiated by modified nano-TiO_2_ in thermal initiation system

**Scheme 4.**
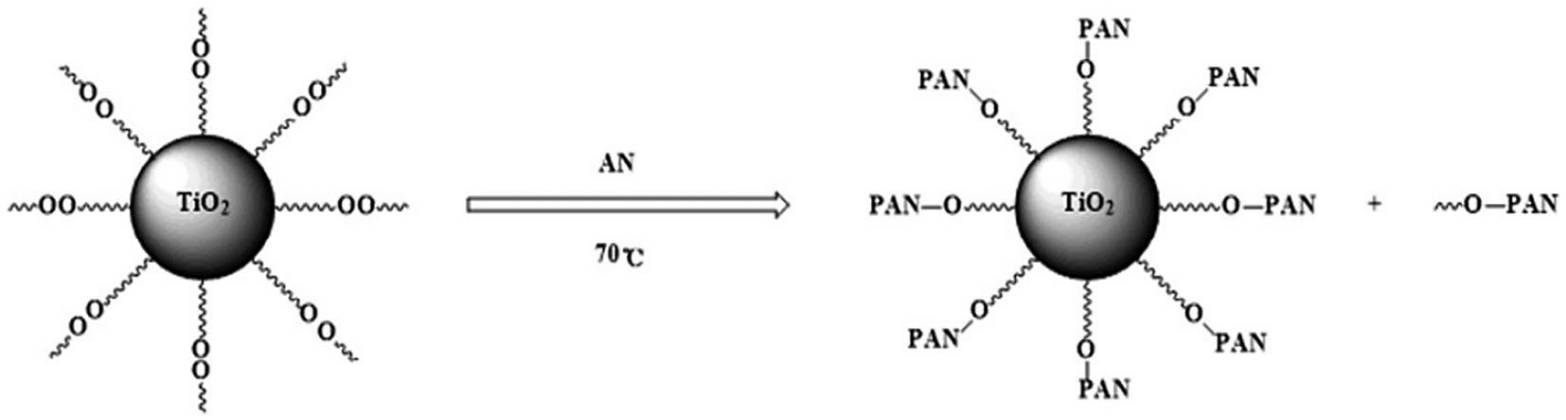
Polymerization of AN initiated by modified nano-TiO_2_ in thermal initiation system.

Modified nano-TiO_2_ was added into a three-neck-flask, stirred at 70 °C for 15 min under purified N_2_ atmosphere.The deionized water and AN in a definite ratio were added into the mixture. The polymerization reaction carried out for 6 h. The unreacted AN and the unattached PAN were separated and refined. The product was dried under vacuum at 60 °C for 24 h. The experiment process was shown as Scheme 4.

#### 2.3.3. Polymerization of AN initiated by modified nano-TiO_2_ in redox initiation system

**Scheme 5.**
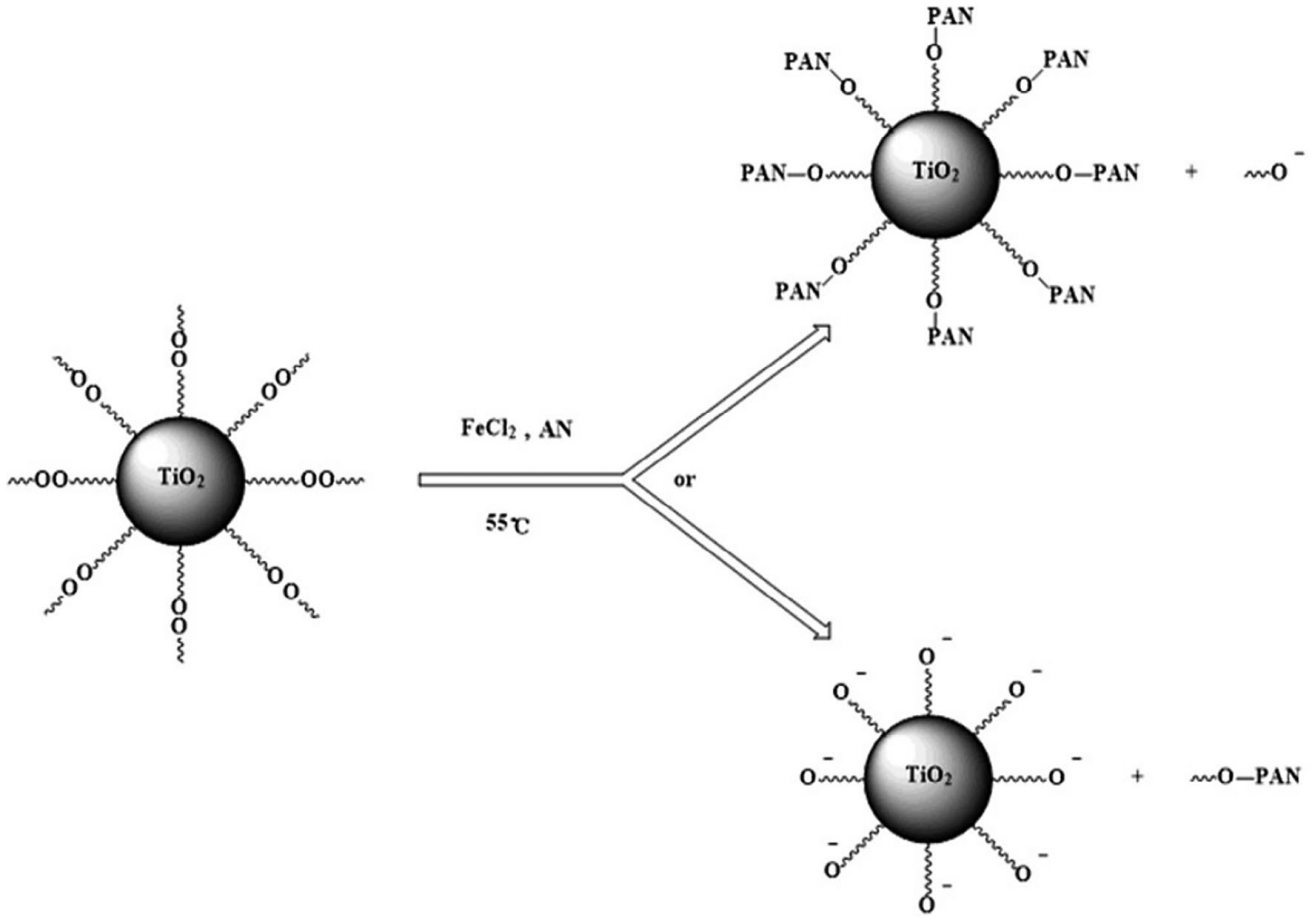
Polymerization of AN initiated by modified nano-TiO_2_ in redox initiation system.

Modified nano-TiO_2_ was added into a three-neck-flask, stirred and heated at 70 °C for 15 min under purified N_2_ atmosphere. The deionized water and AN in a definite ratio were added into the three-neck-flask. Then aqueous solution of ferrous chloride was added. The polymerization reaction carried out for 6 h. The unreacted AN and the uncombined PAN were separated and refined. The product was dried under vacuum at 60 °C for 24 h. The experiment process was shown as Scheme 5.

### 2.4. FTIR characterization

The samples which compressed with KBr were analyzed by Infrared spectrometer (Spectrum One B, PE Company of U.S.A.) at room temperature, the spectral range was 4000–450 cm^−1^, the spectral resolution was 4 cm^−1^.

### 2.5. XPS characterization

XPS spectra were recorded using XPS spectrometer (Perkin Elmer 5100, PE Company of U.S.A.) equipped with an Al K_α_ X-ray source operating at 115 kV and 300 mA. Prior to acquiring XPS spectra, samples were dried in a vacuum oven for 1 day at 100 °C to evaporate residual vapors.

### 2.6. The determination of molecular weight

#### 2.6.1. Cleaving PAN from the TiO_2_-g-PAN

In order to characterize PAN combined with the nano-TiO_2_, the HF solution was used to cleave the TiO_2_-g-PAN. The process was as follows: 2.0 g TiO_2_-g-PAN and 50 mL DMF were mixed for 30 min during the ultrasonic processing, and then 5.0 mL 49 wt% HF solution was added to the mixture, stirred heavily at room temperature for 6 h. The cleaved PAN was obtained.

#### 2.6.2. The determination and calculation of molecular weight

The molecular weight of the free state of PAN and cleaved PAN was calculated using formula (1–3) by means of Ubbelohde viscosity method.

(1) $$\left[\eta \right]=\frac{\sqrt{2(\eta_{\text{sp}}-\ln\eta_{r})}}{\text{c}}={K*M}^{\alpha }$$

(2) $$\eta_{r}=\frac{t}{t_{0}}$$

(3) $$\eta_{\text{sp}}=\eta_{r}-1$$

where $\left[\eta \right]$ – Intrinsic viscosity; $\eta_{\text{sp}}$ – Specific viscosity; *η*_*r*_ – Relative viscosity; *t*_0_ – Solvent efflux time; *t* – Solution outflow time; *c* – Solution concentration; *M* – Viscosity average molecular weight; *K*, *α* – Constant.

### 2.7. The determination of contact angle

Samples were placed in the solution evenly suspended on the surface of a sheet of smooth steel, slowly dried at 50 °C for 24 h to obtain the required test samples. The contact angle surface tension instrument (OCA15EC, Dataphysics Company of Germany) was used to determinate the contact angle. The sample was placed on the test board, and a micro-syringe was hanged over it. Distilled water was squeezed out from the micro-syringe, and then dropped it onto the surface of the sample. Shot the liquid droplets on the surface of the sample by infrared light. Froze the image and measured the contact angle by tangent.

### 2.8. The determination of TG

TG was carried out in N_2_ atmosphere using thermal gravimetric analyzer (STA409PC, Netzsch Company of Germany). About 5–10 mg sample was added into the crucible, the test temperature range was 25–650 °C at a heating rate of 10 °C/min. The whole analysis process was protected by high purity nitrogen with a flow rate of 30 mL/min.

## 3. Results and discussion

### 3.1. FTIR analysis

**Figure 1.**
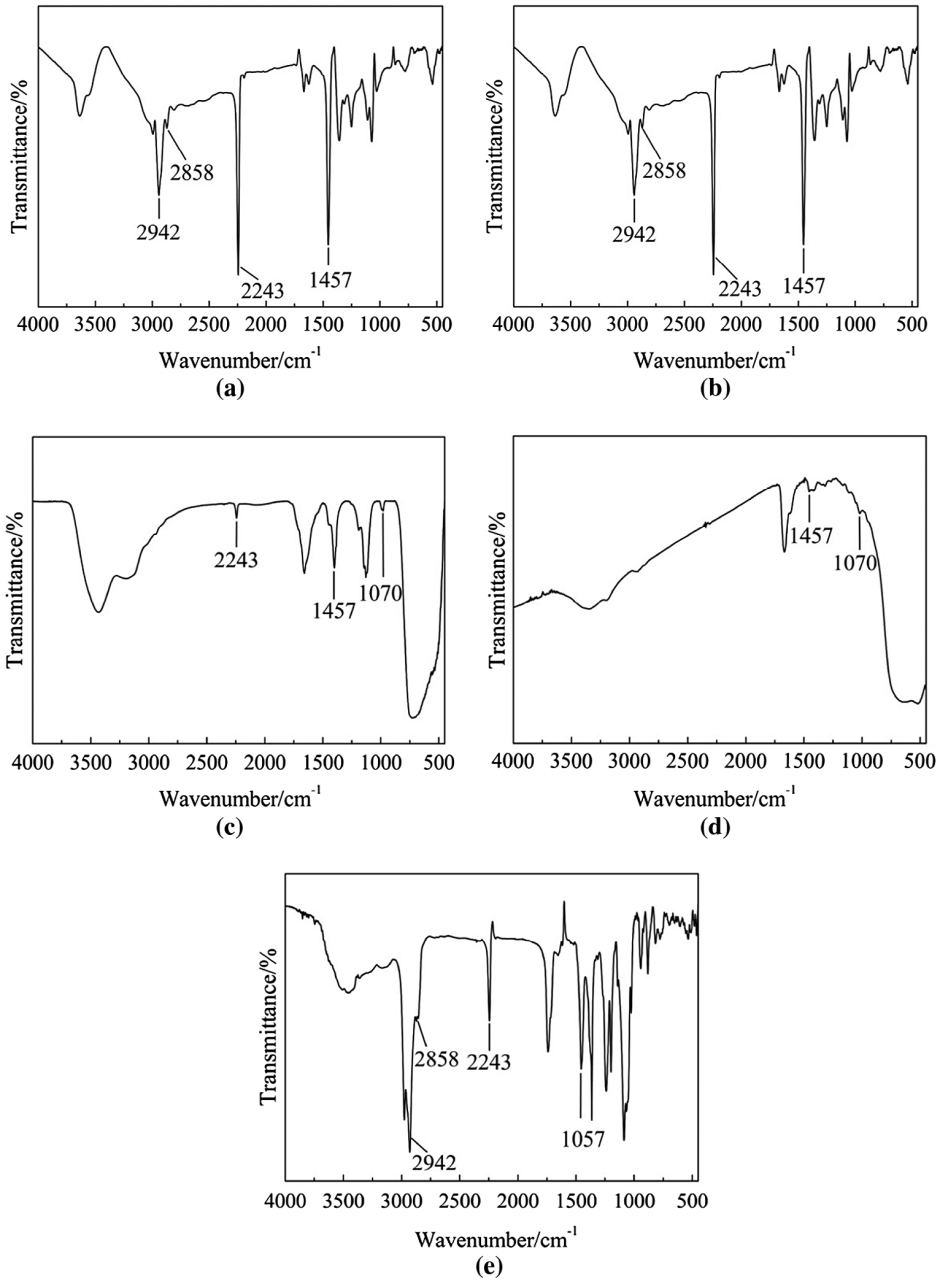
FTIR spectra of products. (a) TBPT as initiator inthermal initiation system. (b) TBPT as initiator in redox initiation system. (c) Modified nano-TiO_2_ as initiator inthermal initiation system. (d) Modified nano-TiO_2_ as initiator in redox initiation system. (e) Washing solution modified nano-TiO_2_ as initiator in redox initiation system.

FTIR spectra of products were shown in Figure 1. From these curves, the peak could be seen at 2942 and 2858 cm^−1^ attributed to the stretching vibration of –CH_2_–, the peak at 1457 cm^−1^ attributed to the bending vibration of C–H, the peak at 1070 cm^−1^ attributed to the stretching vibration of Si–O–C, the peak at 2243 cm^−1^ attributed to the stretching vibration of C≡N.

From the Curve (a) and Curve (b), the peak at 2243 cm^−1^ of C≡N could be seen. It showed that TBPT was able to initiate AN to obtain PAN in thermal initiation system and redox initiation system. The peak at 2243 cm^−1^ of C≡N could be seen in the Curve (c), which indicated the modified nano-TiO_2_ was able to initiate polymerization of AN to produce TiO_2_-g-PAN in thermal initiation system. The Curve (d) and Curve (e) were respectively the FTIR spectra of the solid product and the washing solution of modified nano-TiO_2_ initiating polymerization in redox initiation system. In the Curve (d), the peak at 2243 cm^−1^ could not be seen because TiO_2_-g-PAN was not produced. In the Curve (e), the peak at 2243 cm^−1^ could be seen, which indicated free PAN (the PAN was not bonded on the modified nano-TiO_2_) was produced.

### 3.2. XPS analysis

**Figure 2.**
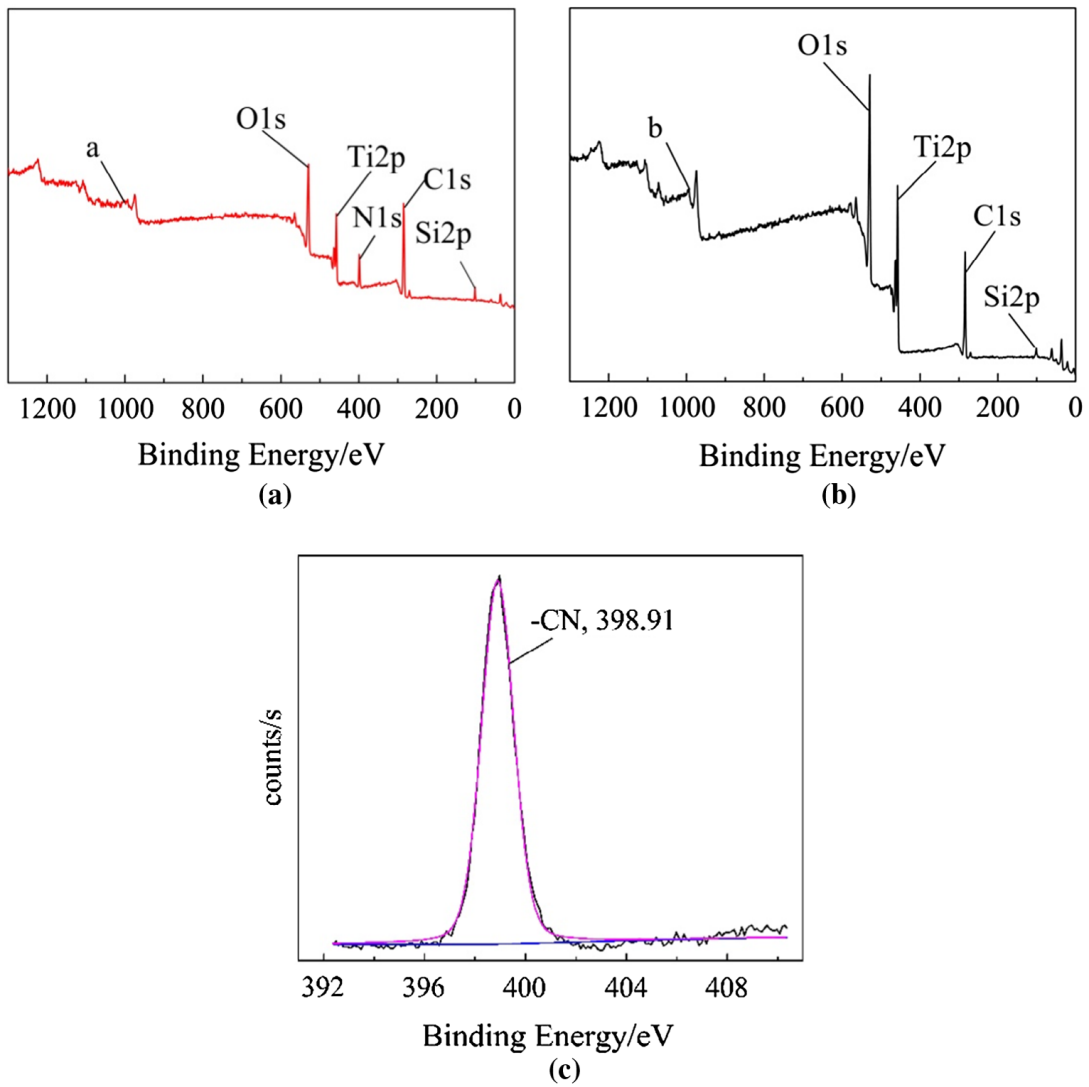
XPS survey spectra of products. (a) Polymerization of AN initiated by modified nano-TiO_2_ in thermal initiation system. (b) Polymerization of AN initiated by modified nano-TiO_2_ in redox initiation system. (c) XPS high resolution N1s spectrum of the Polymerization of AN initiated by modified nano-TiO_2_ in thermal initiation system.

In order to further confirm whether modified nano-TiO_2_ was able to initiate AN polymerization in thermal initiation system and redox initiation system, the products in two initiating approaches were characterized by X-ray photoelectron spectroscopy (XPS). In Figure 2, curve (a) showed that there were Ti, O, C, Si and N. From the high resolution spectrum of N1s in curve (c), there was one kind of chemical state of N: –CN (398.91 eV). Above results indicated there was PAN in the sample. In other words, modified nano-TiO_2_ was able to initiate polymerization of AN to obtain TiO_2_-g-PAN in thermal initiation system. However, Curve (b) showed that there were Ti, O, C, and Si except N, which indicated there was no PAN in the sample. It suggested that modified nano-TiO_2_ cannot initiate polymerization of AN in redox initiation system.

**Figure 3.**
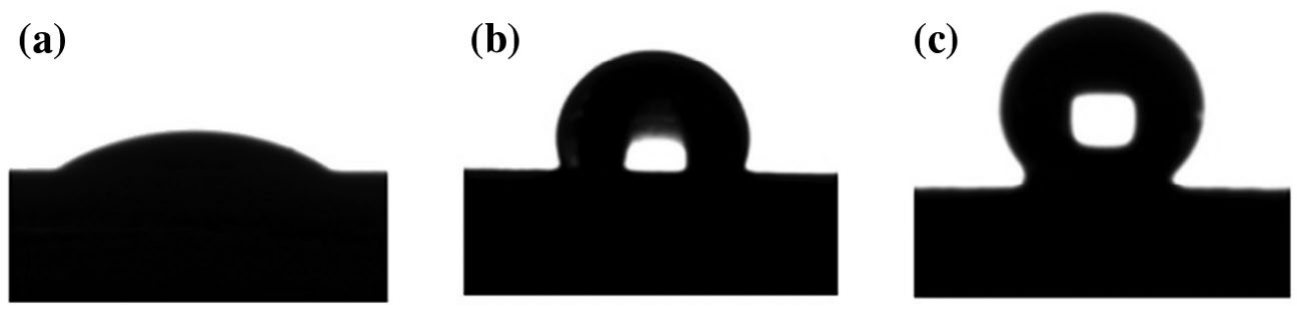
Contact angle analysis. (a) TiO_2_, (b) Modified nano-TiO_2_ and (c) TiO_2_-g-PAN.

**Figure 4.**
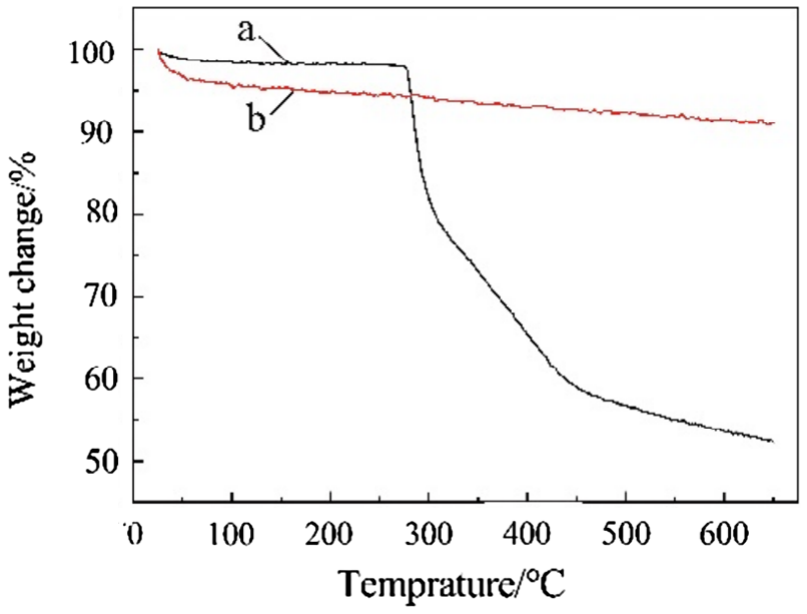
TG spectra. (a) TiO_2_, (b) TiO_2_-g-PAN.

**Scheme 6.**
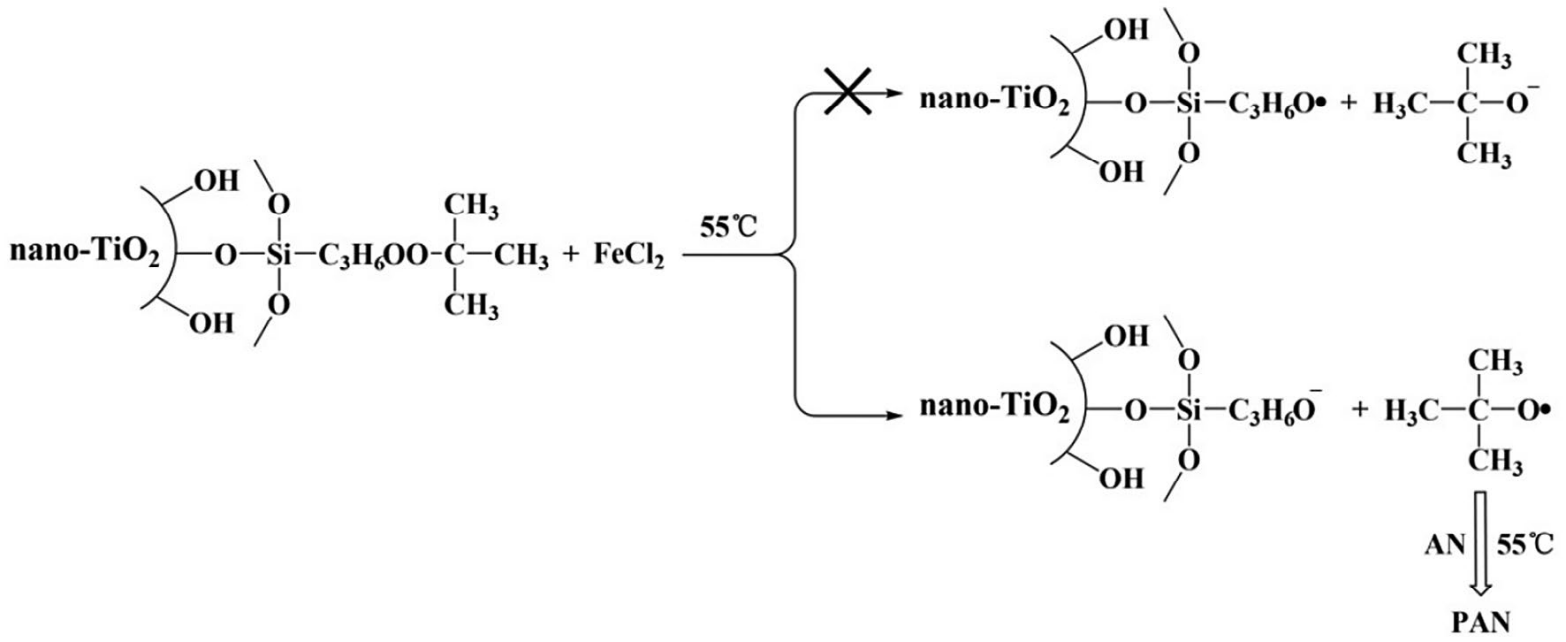
Polymerization of AN initiated by modified nano-TiO_2_ in redox initiation system.

To sum up, the results indicated that modified nano-TiO_2_ produced free radical on the oxygen atom which near the tert butyl group, rather than on the oxygen atom near propyl group in redox initiation system (Scheme 6).

### 3.3. The molecular weight of the products

The molecular weight of obtained PAN from the polymerization of AN was shown in Table 1. In both thermal initiation system and redox initiation system, the molecular weight of four products were approximately 30,000, which indicated that TBPT and modified nano-TiO_2_ (TBPT was bonded to nano-TiO_2_) had similar initiating ability for AN in thermal initiation and redox initiation system.

**Table 1.** The molecular weight of the products.

Initiating approach	Molecular weight of PAN (g/mol)
TBPT in thermal initiation system	3.1 × 10^4^
TBPT in redox initiation system	3.4 × 10^4^
Modified nano-TiO_2_ in thermal initiation system	2.8 × 10^4^
Modified nano-TiO_2_ in redox initiation system	3.0 × 10^4^

### 3.4. Contact angle analysis

Figure 3 was contact angle images of TiO_2_, modified nano-TiO_2_ and TiO_2_-g-PAN. Their Contact angle was 16.25°, 124.36° and 136.42° respectively. TiO_2_-g-PAN was the most hydrophobic among the three samples, and it has a mass of organic component, that means PAN was successfully grafted to the modified nano-TiO_2_.

### 3.5. TG analysis

Figure 4 was TG curves of TiO_2_ and TiO_2_-g-PAN. Curve (a) showed that the weight loss rate of TiO_2_ was 8.9%. In Curve (b) the grafted PAN on the nano-TiO_2_ began to decompose at 290 °C, when the temperature reached 650 °C, the grafted PAN completely decomposed, and the weight loss rate of the TiO_2_-g-PAN was 47.6%. Therefore, the grafting ratio of grafted PAN in TiO_2_-g-PAN was 38.7%.

## 4. Conclusions

The results indicated that TBPT was a novel peroxide silane coupling agent, which could not only initiate the polymerization of AN in thermal initiation system and redox initiation system, but also could be combined with nano-TiO_2_ by covalent bond to prepare the modified nano-TiO_2_. This modified nano-TiO_2_ could also initiate AN to prepare PAN. The results of the polymerization were closely relative to the initiating approach. On one hand, in the products of the thermal initiation system, there were PAN, which attached or unattached to the modified nano-TiO_2_. In other words, in thermal initiation system, both the free PAN and TiO_2_-g-PAN were obtained. On the other hand in redox initiation system the free PAN was obtained, but TiO_2_-g-PAN was not. The grafting ratio of grafted PAN in TiO_2_-g-PAN was 38.7%. The molecular weight of PAN produced in four methods was at range of 2.8 × 10^4^–3.4 × 10^4^.

## Disclosure statement

No potential conflict of interest was reported by the authors.

## Funding

This work was financially supported by the National Natural Science Foundation of China [grant number 21376127], [grant number U1162123]; the National Natural Science Foundation of China, the Youth Science Fund project of National Science Fund [grant number 51103076].
